# A goodness‐of‐fit test for occupancy models with correlated within‐season revisits

**DOI:** 10.1002/ece3.2292

**Published:** 2016-07-05

**Authors:** Wilson J. Wright, Kathryn M. Irvine, Thomas J. Rodhouse

**Affiliations:** ^1^ Department of Mathematical SciencesMontana State UniversityBozemanMontana59717; ^2^ Northern Rocky Mountain Science CenterU.S. Geological SurveyBozemanMontana59715; ^3^ Upper Columbia Basin NetworkNational Park ServiceBendOregon97702

**Keywords:** Acoustic surveys, bats, independence assumption, join count, Markov occupancy model, model assessment, monitoring, serial correlation

## Abstract

Occupancy modeling is important for exploring species distribution patterns and for conservation monitoring. Within this framework, explicit attention is given to species detection probabilities estimated from replicate surveys to sample units. A central assumption is that replicate surveys are independent Bernoulli trials, but this assumption becomes untenable when ecologists serially deploy remote cameras and acoustic recording devices over days and weeks to survey rare and elusive animals. Proposed solutions involve modifying the detection‐level component of the model (e.g., first‐order Markov covariate). Evaluating whether a model sufficiently accounts for correlation is imperative, but clear guidance for practitioners is lacking. Currently, an omnibus goodness‐of‐fit test using a chi‐square discrepancy measure on unique detection histories is available for occupancy models (MacKenzie and Bailey, *Journal of Agricultural, Biological, and Environmental Statistics*, 9, 2004, 300; hereafter, MacKenzie–Bailey test). We propose a join count summary measure adapted from spatial statistics to directly assess correlation after fitting a model. We motivate our work with a dataset of multinight bat call recordings from a pilot study for the North American Bat Monitoring Program. We found in simulations that our join count test was more reliable than the MacKenzie–Bailey test for detecting inadequacy of a model that assumed independence, particularly when serial correlation was low to moderate. A model that included a Markov‐structured detection‐level covariate produced unbiased occupancy estimates except in the presence of strong serial correlation and a revisit design consisting only of temporal replicates. When applied to two common bat species, our approach illustrates that sophisticated models do not guarantee adequate fit to real data, underscoring the importance of model assessment. Our join count test provides a widely applicable goodness‐of‐fit test and specifically evaluates occupancy model lack of fit related to correlation among detections within a sample unit. Our diagnostic tool is available for practitioners that serially deploy survey equipment as a way to achieve cost savings.

## Introduction

Occupancy modeling is a widely used analytical framework for making inferences about distributions of threatened and elusive plant and animal populations (Bailey et al. 2014). Occupancy models can provide unbiased estimates of species occurrence, even when the probability of detection is less than one (MacKenzie et al. 2006). Additionally, occupancy estimates can be more informative and practically obtained than other state variables used to address a wide variety of research and monitoring goals (e.g., Jones 2011). Many research and monitoring programs deploy automated recording devices for consecutive days or nights to detect species of interest (e.g., Acevedo and Villanueva‐Rivera 2006; Hines et al. 2010; Furnas and Callas 2015; Loeb et al. 2015). While this strategy can reduce costs per unit of survey effort, it also can lead to violation of a fundamental occupancy model assumption that observations within a detection history (i.e., repeat visits to a sample unit within a season) are independent. The ubiquity of occupancy studies that utilize within‐season replicates that are measured in spatial or temporal proximity creates a need for tools that can assess model lack of fit from correlated detections.

Making reliable inferences from models requires a critical assessment of implicit model assumptions and evaluation of model–data agreement. In general, assessment and validation of occupancy models have received less attention in the literature (e.g., MacKenzie and Bailey 2004) than has the development of more complex models (MacKenzie et al. 2006; Royle and Dorazio 2008; Hines et al. 2010). A goodness‐of‐fit test based on a Pearson's chi‐square test statistic using unique detection histories has been shown to be an effective tool at diagnosing lack‐of‐fit model due to nonindependent sample units or missing detection probability covariates (MacKenzie and Bailey 2004). Further, Bayesian posterior predictive checks and area under the curve values have been used in model validation (Zipkin et al. 2012; Rodhouse et al. 2015; Kery and Royle 2016). However, to our knowledge, the performance of tools to identify temporal or spatial correlation among observations used for estimating detectability has not been investigated thoroughly. Additionally, despite the MacKenzie–Bailey test being the standard tool for assessing model fit in an occupancy framework, it does not appear to have been widely used [e.g., MacKenzie et al. (2002) cited 1353 times whereas MacKenzie and Bailey (2004) cited only 178 times; Web of Science [wokinfo.com] accessed on 31 May 2016]. Frequently, information theoretic measures, such as Akaike's (AIC), are used to compare, in a relative way, information content of different occupancy models, but this approach does not actually assess how well the suite of candidate models fit the data (Burnham and Anderson 2002).

As occupancy models are frequently utilized to guide management decisions for endangered or threatened species (Jones 2011), unrealistic models that lead to incorrect inferences have the potential to be extremely costly to conservation efforts. Misleading inferences are possible if the statistical model used does not adequately describe the observed data. Failing to appropriately model correlation among sample units results in inaccurate standard errors (MacKenzie and Bailey 2004), while unmodeled correlation among revisits can lead to biased occupancy estimates (Hines et al. 2010) or can reduce power to detect changes in occupancy over time (Whittington et al. 2015). Explicitly modeling detection‐level correlation as a first‐order Markov structure or continuous process is some of the proposed solutions when serial revisits are correlated (e.g., Hines et al. 2010; Guillera‐Arroita et al. 2011; Charbonnel et al. 2014; Whittington et al. 2015). An alternative approach is to account for correlated revisits prior to an analysis by aggregating observations until they can be considered independent. However, we are unaware of any tests or procedures that specifically evaluate whether there is evidence of correlation within survey site detection histories or that may be left unaccounted for after fitting a model. Here, we expand the occupancy modeling tool kit by providing an alternative procedure for practitioners to use for assessing lack of independence when detection data are gathered in close geographic or temporal proximity.

We motivate our work by the within‐season revisit design proposed by Loeb et al. (2015) for the North American Bat Monitoring Program (NABat) where multiple (2–4) bat detectors deployed at different locations over four consecutive nights within each areal sample unit is recommended. Under this framework, the detection or nondetection of a species during one night at a single detector is considered a replicate survey of the sample unit (or revisit) for occupancy analysis. Although we focus on the deployment of bat detectors over consecutive nights, we note that study designs that generate serial encounter histories are widespread for many taxa (e.g., Hines et al. 2010). However, the bat monitoring scenario is especially useful because the cost‐saving benefit of serial deployment is great. The cryptic and wide‐ranging movements of bats make them particularly difficult to survey without assistance from recording devices (Hayes et al. 2009); deploying these devices over days and weeks can generate large amounts of data, offsetting the initial investment in travel and other deployment costs. There are many applications where bat detectors have been deployed over consecutive nights or simultaneously across space as replicates within areal sample units to study habitat associations, bat activity patterns at wind farms, and for population monitoring (e.g., Gorresen et al. 2008; Fischer et al. 2009; Weller and Baldwin 2012; Rodhouse et al. 2015). Because of the growing number and novelty of the conservation threats facing bats from disease (Frick et al. 2010), wind power developments (Hayes 2013), and climate change (Sherwin et al. 2013), developing appropriate statistical methods and model assessment diagnostics for data arising from acoustic‐based bat surveys is increasingly urgent.

We develop a novel goodness‐of‐fit test based on a chi‐square discrepancy measure using join counts adapted from spatial statistics. We compare our join count chi‐square test to the MacKenzie–Bailey test that is currently available to evaluate the fit of occupancy models. Our test is similar to the MacKenzie–Bailey test because it is based on a chi‐square discrepancy measure, but aggregates over sample units by the join count statistic commonly used for assessing spatial correlation in binary lattice data (Schabenberger and Gotway 2005) instead of unique detection histories. Our approach provides a tool to specifically assess the occupancy model assumption of independent observations from a sample unit within a season. We investigate how the number of spatial and temporal replicates within a sample unit (within‐season revisit design structure) and strength of serial correlation among temporal replicates influence the ability of these tests to detect whether a Markov model (also referred to as the “trap response” model from Hines et al. 2010) or single‐season occupancy model (MacKenzie et al. 2006) is adequate for drawing ecological inferences via a simulation study. We also demonstrate the use of these model assessment tools with empirical data collected during a pilot study for NABat. Our approach illustrates that sophisticated models do not guarantee adequate fit to real data, underscoring the importance of model assessment. Computer code to implement our join count chi‐square test is available in Supporting Information (Data S1).

## Methods

### Occupancy models

We investigated two occupancy models: a single‐season occupancy model (MacKenzie et al. 2006) and a single‐season model which incorporated a first‐order Markovian process for detections at consecutive replications (based on the “trap response” model from Hines et al. 2010). We consider detection history matrices composed of observations from temporal and spatial replicates within a sample unit. Using both types of within‐unit replication has been proposed for the NABat program (Loeb et al. 2015), but is not typical for single‐season occupancy models (but see Whittington et al. 2015) and not widely investigated within an occupancy modeling framework. Under the assumption that spatial and temporal revisits are independent and exchangeable, an occupancy model incorporating both types of replication is equivalent (mathematically) to a single‐season occupancy model. We chose to retain three indices in our notation below to more easily distinguish spatial (e.g., detectors at different locations within a sample unit) from temporal (e.g., a survey night) revisits within a detection history for a sample unit when describing our join count statistics.

Under this framework, observation *Y*_*ijk*_ was a binary response (detection = 1, nondetection = 0) for sample unit *i* with *i* = 1, …, *N*, spatial replicate *j* with *j* = 1, … *J*, and temporal replicate *k* with *k* = 1, …, *K*. Under the standard, single‐season occupancy model, detections occur with probability *p* conditional on the sample unit being occupied (*z*_*i*_ = 1) which occurs with probability *ψ*. Estimates of detection (*p*) and occupancy (*ψ*) probabilities are simultaneously obtained using revisits to sampling units (MacKenzie et al. 2006). Covariates can be included in this model using a logit link which allows *p* and (or) *ψ* to vary as follows, logit(*ψ*_*i*_) = *X*_*i*_ *β* and logit(*p*_*ijk*_) = *V*_*ijk*_ *α*, where *X*_*i*_ and *V*_*ijk*_ are occupancy and detection covariate vectors, respectively, while *β* and *α* are coefficient vectors. Under this model, spatiotemporal observations (*Y*_*ijk*_) are assumed independent draws from a zero‐inflated binomial distribution (hereafter, “basic” occupancy model).

The second model we utilized (hereafter, “Markov” model) is an extension of the basic model which incorporates different detection probabilities based on whether the previous temporal revisit yielded a detection. As with the basic model, occupancy and detection‐level covariates can be incorporated using a logit link. The Markov model incorporates a first‐order Markov‐structured, detection‐level covariate. The model specifies the probability of detecting a species as *p*_1_ if the previous survey resulted in a detection and *p*_0_ if there was not a detection in the previous survey. Based on the idea that nightly bat activity drives changes in detection, when Markov models were fit, we assumed a detection probability of *p*_mean_ =*p*_0_/(*p*_0_ + (1 − *p*_1_)) if the survey was the first temporal replicate because this would be the equilibrium probability for a randomly selected night (Hines et al. 2010).

Although the Markov model can also be used to model correlation from spatial replication (e.g., consecutive trail segments from Hines et al. 2010), we chose to simplify the problem to only temporal correlation for purposes of method development. Based on knowledge about bat biology and our own exploratory analyses, we assumed that bat acoustic survey data were consistent with a Markov model in which consecutive temporal observations were potentially correlated, but observations from spatial replicates were independent when spaced minimum distances apart within areal sample units as enforced via a survey protocol (e.g., kilometers) (Appendix S1). Several studies have identified the potential for “runs” in bat activity to occur over consecutive nights as a result of weather fronts (Hayes 1997; Fischer et al. 2009). In general, temperate zone bats are nonterritorial central‐place foragers with large home ranges (Pierson 1998; Daniel et al. 2008; Popa‐Lisseanu et al. 2009; Rainho and Palmeirim 2011), supporting the assumption that spatial replicates within occupied areal units all have nonzero probability of species detection. Although here we make the assumption that spatial replicates are independent throughout, different biological and sampling design scenarios could yield different patterns of spatiotemporal correlation (e.g., Charbonnel et al. 2014; Whittington et al. 2015) and should be investigated further (e.g., with multiscale model by Nichols et al. 2008).

To fit both models, we used maximum‐likelihood estimation (MLE) in Program R (R Core Team 2015). Likelihood functions were written for both occupancy models, and MLE solutions for the parameters were found using the *optim* function with initial values for all parameters equal to zero on the logit scale (corresponding to a probability of 0.5). Asymptotic 95% confidence intervals for all parameters were found using the associated Hessian matrix given by the *optim* function. Confidence intervals for *p* and *ψ* were estimated on the logit scale and back‐transformed to the probability scale.

### Mackenzie–Bailey test

We explored a goodness‐of‐fit test based on a Pearson's chi‐square test statistic based on the counts of unique detection histories (MacKenzie and Bailey 2004) as one tool to check for possible violation of the independence assumption. As pointed out by Kery and Royle (2016), the MacKenzie–Bailey test utilized one method to aggregate the data (by unique detection histories), but other data aggregations, such as row or column sums from the detection history matrix, could be used. The MacKenzie–Bailey test statistic was calculated using the observed number of sample units with detection history *h* (*O*_*h*_) and the expected number of sample units with detection history *h* (*E*_*h*_) given parameter estimates from a fitted model (here, the basic or Markov) and the number of sample units visited. Let *y*_*i*.._ denote the vector of observations from sample unit *i* (i.e., (*y*_*i*11_, *y*_*i*12_, …,*y*_*i*1*K*_, *y*_*i*21_, …, *y*_*iJK*_)); *H* the set of all possible detection histories observed for *y*_*i*…_ For example, with *J* = 2 and *K* = 2, one possible outcome in the set *H* could be *h* = 0101, with elements *h*_11_ = *h*_21_ = 0 and *h*_12_ = *h*_22_ = 1. *E*_*h*_ and *O*_*h*_ are defined as follows: $O_{h}=\sum_{i=1}^{N}I\left(y_{i..}=h\right)$ and

$$E_{h}=\sum_{i=1}^{N}{\hat{\psi }}_{i}\times \hat{\mathrm{Pr}}\left(y_{i..}=h|z_{i}=1\right)+\left(1-{\hat{\psi }}_{i}\right)I\left(h=0\right),$$

where *I*(*h* = 0) is an indicator if *h* was all zeroes or not, and

$$\hat{\mathrm{Pr}}\left(y_{i..}=h|z_{i}=1\right)=\prod_{j=1}^{J}\prod_{k=1}^{K}{\hat{p}}_{ijk}h_{jk}+\left(1-{\hat{p}}_{ijk}\right)\left(1-h_{jk}\right).$$

Correction statement: [Correction added on 15 July 2016: One of the equations was previously incorrect and has been amended in this version.]

In words, the probability of a given detection history vector for sample unit *i* given occupied is based on estimated probabilities ${\hat{p}}_{ijk}$ multiplied by the corresponding detection or nondetection element *h*_*jk*_ within the vector *h*. A computational efficiency was gained because only expected counts for observed combinations of detection/nondetections were needed in calculations (MacKenzie et al. 2006, Pp. 110–113).

If the survey design was not balanced (i.e., *J* and *K* were not equal for every sample unit), these values were determined for each cohort (c), where a cohort was all the sample units with the same pattern of missing observations (MacKenzie and Bailey 2004). The MacKenzie–Bailey test was based on a Pearson's chi‐square test statistic or a chi‐square discrepancy measure (Kery and Royle 2016):

$$\chi^{2}=\sum_{c}\sum_{h_{c}}\frac{{\left(O_{h_{c}}-E_{h_{c}}\right)}^{2}}{E_{h_{c}}}.$$

*P*‐values were found using a parametric bootstrap approach (MacKenzie and Bailey 2004) with 500 replications per test. The *P*‐value was the proportion of generated datasets under the fitted model which resulted in a Pearson's chi‐square test statistic greater than or equal to the observed test statistic calculated from empirical data. Small *P*‐values suggest the observed data were inconsistent with the fitted model (lack of fit). R code to implement this test for the models we investigated is available in Supporting Information (Data S1).

### Permutation join count test

We exploited the join count, a spatial statistic, for assessing whether there was evidence for lack of independence among observations within the detection history matrix. Join count tests were developed for evaluating spatial correlation among binary data collected over a lattice (Schabenberger and Gotway 2005). We modify the join count statistic by defining a “neighborhood” of temporal observations within a sample unit. We explored two different neighbor definitions: (1) all temporal observations (*k* = 1, …, *K*) at a single spatial replicate *j* within the same sample unit *i* and (2) observations from consecutive temporal revisits indexed by *k* − 1*, k, k* + 1 at a single spatial replicate *j* within the same sample unit *i*. Under definition (2), temporal observation 1 and *K* have only one neighbor each, temporal observation 2 and *K *− 1, respectively, while the remaining temporal observations have two neighbors each. Visual representations of these neighborhood definitions for two example within‐season revisit designs are shown in Appendix S2. The two different neighbor definitions illustrate that even though we focused on Markovian dependence among detections for this study, our test is applicable for other spatial and (or) temporal processes that lead to correlated detections. The join count statistic was then determined by counting the number of neighbor pairs (using either definition above) with species detections within each sample unit *i* (number of pairs with *Y*_*ijk*_ = 1 and *Y*_*ijk*’_ = 1, where these observations are neighbors; i.e., “black–black” joins), referred to as BB statistic.

*P*‐values associated with the BB statistic, in a spatial analysis context, are found using a permutation test and calculated as the proportion of permuted datasets resulting in a total number of joins (i.e., sum the BB statistics over all sample units) larger than the total number of joins observed. The null distribution for this permutation test assumes that there was no correlation among observations. We generated permuted datasets through a constrained randomization that rearranged the observations (zeros and ones) in the detection history matrix using the sample units with at least one detection (naive occupancy). In other words, the randomization constraint shuffled observations within and across naively occupied sample units and then the test statistic (total joins) was calculated for these permuted datasets. The randomization constraint avoided possible diffusion of detection/nondetections across a greater number of sample units (including to some that are not occupied) which would lead to an observed test statistic consistently in the tails of the permutation distribution. In other words, without the constraint, one would incorrectly assume there was temporal correlation in a dataset.

We used this method as an exploratory tool (prior to fitting any model). The typical join count permutation test procedure assumed that there was no heterogeneity in the probability of detection (*p*_*ijk*_ = *p* for all *i*,* j*,* k*). Therefore, this test was limited because detection covariates were not considered and there was no ability to assess evidence of correlation after fitting a model. To perform this test, we used the spdep package in R (Bivand and Piras 2015).

### Join count chi‐square test

To address the limitations of the join count test, we developed a novel test procedure utilizing a chi‐square test for the observed and expected number of join count (BB) values within sample units. Our procedure can be used after fitting any occupancy model which may include detection‐level covariates, such as a first‐order Markov‐structured covariate, or possibly other model extensions. We used the same neighbor definitions as described in the preceding section. However, we compared the observed count of sample units with a particular number of joins (based on either neighbor definition: *O*_BB_) to the expected count (*E*_BB_) based on the estimated detection and occupancy probabilities from a fitted model. Then, similar to the MacKenzie–Bailey test, *O*_BB_ and *E*_BB_ were found for the set of possible number of joins (BB) within a detection history, *H* (again *h* denotes a possible outcome in the set *H*), with

$$O_{\mathrm{BB}}=\sum_{i=1}^{N}I\left(\text{joins}\left(y_{i..}\right)=\text{BB}\right),$$

and

$$E_{\mathrm{BB}}=\sum_{h}E_{h}\times I\left(\text{joins}\left(h\right)=\text{BB}\right),$$

where *I*(joins(*h*) = BB) is an indicator if the number of joins in *h* is equal to BB and *E*_*h*_ is calculated in the same way as performed for the MacKenzie–Bailey test.

The join count chi‐square test statistic equals $\sum_{c}\sum_{{\mathrm{BB}}_{c}}{\left(O_{{\mathrm{BB}}_{c}}-E_{{\mathrm{BB}}_{c}}\right)}^{2}/E_{{\mathrm{BB}}_{c}}$. The summation over cohorts modifies the statistic to accommodate an unbalanced survey design, as performed for the MacKenzie–Bailey test. We provide example calculations for our join count chi‐square test using a simple dataset for both the basic and Markov models in Appendix S2.

All *P*‐values associated with the join count chi‐square test were also found using a parametric bootstrap approach and calculated as the proportion of bootstrapped test statistics larger than the observed test statistic. A small *P*‐value suggested that observed data were inconsistent with a fitted model based on the BB counts, whereas a large *P*‐value indicated the observed BB counts were consistent with those generated from the fitted model.

The main distinction between the MacKenzie–Bailey test and our novel join count chi‐square test was the data aggregation used within the chi‐square discrepancy measure. The MacKenzie–Bailey test focused on patterns of nondetections/detection, a more omnibus approach. Comparatively, the join count chi‐square test examined whether the number of joins (neighbors with both responses of 1) within sample units observed in the data was likely under a fitted model. In this way, the join count chi‐square test provides a way to specifically evaluate whether there is evidence of additional correlation after fitting a model. R code to perform this test for the models we investigated is available in the Supporting Information (Data S1) and could be modified to accommodate other occupancy model extensions.

### Simulation study

The MacKenzie–Bailey, permutation join count, and our new join count chi‐square tests (using both neighbor definitions) were applied to simulated datasets under four different scenarios. For all scenarios, we assumed a sample size of 50 (*i *=* *1,…,50) and that all sample units had the same number of spatial and temporal replicates within a season. The four within‐season revisit designs were as follows: a single spatial replicate (*J* = 1) with 16 temporal revisits (*K* = 16); four spatial replicates (*J* = 4) with four temporal revisits (*K* = 4); a single spatial replicate (*J* = 1) with eight temporal revisits (*K* = 8); and four spatial replicates (*J* = 4) with two temporal revisits (*K* = 2). Of these scenarios, the first two have 16 total revisits per sample unit and the second two have eight total revisits per sample unit.

We assumed that spatial replicates were independent of one another and varied the strength of correlation between consecutive temporal observations within a sample unit. For each revisit schedule, we simulated data under five different *p*_0_/*p*_1_ combinations: 0.5/0.5, 0.4/0.6, 0.3/0.7, 0.2/0.8, and 0.1/0.9 with the probability of detection always equal to *p*_mean_ for the first temporal replicate. Note that the difference in detection probabilities (*p*_1_ − *p*_0_) increased as the correlation among detections increased, but that for each combination, *p*_mean_ was equal to 0.5. The *p*_0_/*p*_1_ combination of 0.5/0.5 represents a scenario of no correlation among temporal replicates as the probability of detection does not depend on whether the previous observation resulted in a detection or not.

For each within‐season revisit design scenario and *p*_0_/*p*_1_ combination, 500 datasets were simulated. The permutation‐based join count test was performed on each simulated dataset with 999 permuted (using the constrained randomization procedure) datasets per test. Then, the Markov and basic occupancy models (no covariates) were fit to each simulated dataset. For all scenarios and *p*_0_/*p*_1_ combinations, the Markov model was always consistent with how the data were simulated including the assumption that the detection probability for the initial temporal replicates was *p*_mean_. If a model did not converge for a particular dataset (i.e., error from the *optim* function or the inverse of the Hessian was not positive definite), the tests using that model fit were discarded. Using both fitted models, we then performed the MacKenzie–Bailey test and join count chi‐square test (using both neighborhood definitions for every simulated dataset).

We calculated the proportion of the 500 simulated datasets which resulted in *P*‐values less than 0.05 for all tests, within‐season revisit scenarios, and *p*_0_/*p*_1_ combinations as a summary measure. This measure could be considered an estimate of test *power* for appropriately detecting this particular form of correlation after model fitting. For example, fitting the basic occupancy model to data generated with *p*_0_/*p*_1_ = 0.5 (no correlation) should result in only 5% of the 500 datasets returning tests with *P*‐values < 0.05 (using a fixed cutoff *α* = 0.05), if the test displays the nominal rate. However, for the scenarios that generate datasets with serial correlation (*p*_0_/*p*_1_ ≠ 0.5), the MacKenzie–Bailey and join count chi‐square tests should detect inadequacy of the basic model, ideally, resulting in power increasing as the strength of serial correlation increases. On the other hand, MacKenzie–Bailey and join count chi‐square tests should indicate for the majority of simulated datasets adequacy of the Markov model as the data were generated under that model when *p*_0_/*p*_1_ ≠ 0.5, again displaying nominal test size.

### Bat acoustic survey data

To demonstrate application of these model assessment procedures, we used call file data identified to bat species collected during surveys on U.S. Fish and Wildlife refuges across Washington, Oregon, and Idaho (Barnett 2014). For this dataset, following a regional sampling design (Rodhouse et al. 2011, 2015) that has subsequently been expanded to support continent‐wide monitoring (Loeb et al. 2015), a sample unit (*N* = 57) was a 10 × 10 km grid cell and spatial replicates (*J* = 1,…,4) were stations where a detector was placed at a point location within a sample unit. Some sample units had spatial replicates that were surveyed over the same nights, but others had detectors deployed over different consecutive nights. Recorded calls were classified to species using commercial software (Sonobat 3; http://www.sonobat.com/). We constructed a binary response variable, detected or not detected (1/0), within each station/night combination.

We included only two species, the big brown bat (*Eptesicus fuscus*; hereafter EPFU) and the hoary bat (*Lasiurus cinereus*; hereafter LACI) in this study, although many more species were recorded during the surveys (Appendix S1). These species are common and widespread and provided rich detection history patterns for exploration. For both species, we used a subset of the available data such that all sample units had at most four detectors (spatial replicates) and at most four consecutive nights (temporal replicates) per detector. We modeled bat occupancy as a function of whether the areas surrounding the sample unit included land used for agriculture or not and bat detectability as a function of the type of water source near a detector (factor with four levels: creeks and streams, lakes and ponds, marshes and sloughs, and other (typically man‐made) water features) and Julian date.

## Results

### Simulation study

We present results based only on model fits that appeared to have adequately converged. Lack of convergence occurred when the *optim* function reported a Hessian matrix whose inverse was not positive definite. Convergence issues occurred most frequently for Markov models when serial correlation was high (i.e., large difference in *p*_0_ and *p*_1_). The problem was most severe for the scenario with a single spatial replicate and 16 temporal replicates and *p*_1_ − *p*_0_ = 0.8; almost half (236) of the Markov models failed to converge. For the remaining scenarios, the issue was much less prevalent. We did not explore ways to resolve this issue for the simulation study, but different initial values or settings for the numerical algorithm could improve computations.

### Occupancy and detection estimation

For converged models, estimates of occupancy (*ψ*) and detection (*p*) from the Markov model appear unbiased and 95% confidence interval coverages were close to nominal (i.e., 95% of the intervals contained the true value) for almost every combination of serial correlation and spatiotemporal replicates (Table C1 in Appendix S3). The one exception was with one spatial and eight temporal replicates with *p*_1_ − *p*_0_ = 0.8; occupancy estimates were slightly biased, and coverage was only 91.2%. Estimates of *p*_0_ and *p*_1_ using the Markov model appeared unbiased for all spatiotemporal replicate and strength of serial correlation combinations (Tables C2 and C3 in Appendix S3). The confidence intervals for *ψ* and *p* were wider using the Markov model compared to the basic model (Tables C2, C3, and C4 in Appendix S3). This finding is consistent with the understanding that confidence intervals will be too narrow when correlation is unaccounted for within a model.

For the basic model, occupancy estimates were unbiased when correlation (*p*_1_ − *p*_0_) among detections was low, but bias increased as correlation increased (Table C1 in Appendix S3). This issue was not as pronounced if independent spatial replicates were available (as well as temporal replicates), but became more severe when there were no independent within‐season observations for a sample unit. For instance, with the highest level of correlation, we investigated (*p*_1_ − *p*_0_ = 0.8) the occupancy (*ψ*) 95% confidence interval coverage rates were 0.944 and 0.908 for scenarios with four spatial replicates (four and two temporal revisits, respectively) while the occupancy (*ψ*) coverage rates dropped to 0.758 and 0.226 for the same level of correlation when only a single spatial replicate was used (16 and 8 temporal revisits, respectively; Table C1 in Appendix S3).

### Comparison of model assessment tests: Mackenzie–Bailey, permutation join count, and join count chi‐square tests

**Figure 1:**
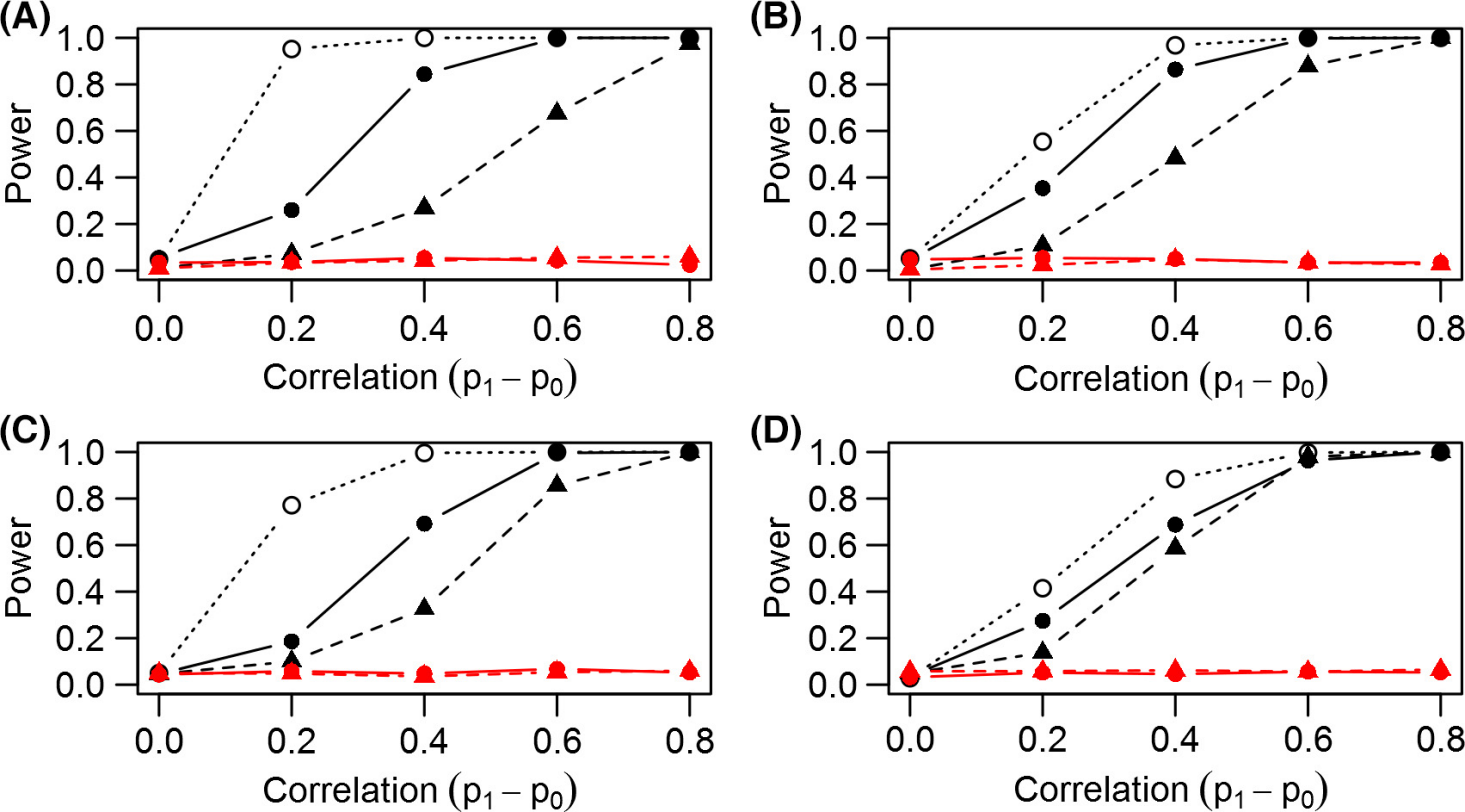
Defining neighbors as all temporal observations within a spatial replicate, the proportion of tests on simulated datasets with *P*‐values less than 0.05 (denoted as power) for scenarios with four spatial, four temporal (A); one spatial, 16 temporal (B); four spatial, two temporal (C); and one spatial, eight temporal (D) replicates versus correlation (difference in detection probabilities). Different line types correspond to different tests with solid lines for the novel join count chi‐square test and dashed lines for the MacKenzie–Bailey test. Color distinguishes the two models: black lines for tests using the basic model and red for Markov model. The permutation‐based join count test is shown with the open circles and dotted line. The plot shows join count chi‐square test detects model inadequacy at a higher rate than the standard MacKenzie–Bailey test when using the basic model under all four scenarios. The Markov model displays nominal test size (power = 0.05) for datasets with serially correlated detections.

**Figure 2:**
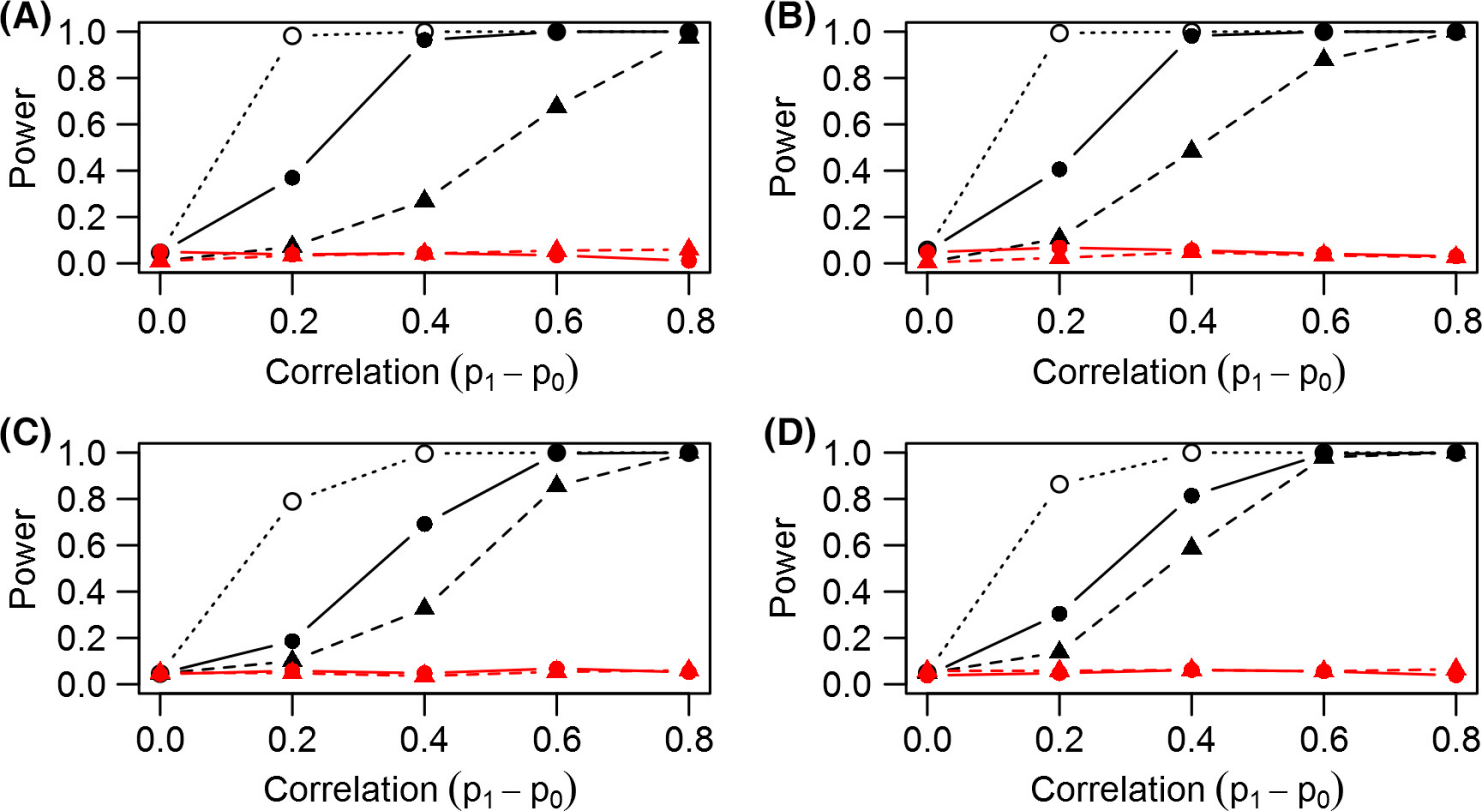
Defining neighbors as consecutive temporal observations within a spatial replicate, the proportion of tests on simulated datasets with *P*‐values less than 0.05 (power) for scenarios with four spatial, four temporal (A); one spatial, 16 temporal (B); four spatial, two temporal (C); and one spatial, eight temporal (D) replicates versus correlation (difference in detection probabilities). Different line types correspond to different tests: solid lines for the novel join count chi‐square test; dashed lines for the MacKenzie–Bailey test; permutation join count test shown by open circles and dotted line. Color distinguishes the two models: black lines for tests using the basic model and red for Markov model. The plot shows the join count chi‐square test using this neighbor definition also detects model inadequacy at a higher rate than the MacKenzie–Bailey test when using the basic model under all four scenarios. The Markov model displays nominal test size (power = 0.05) for datasets with serially correlated detections.

We consistently found that the join count chi‐square test had greater ability to correctly identify unaccounted for correlation (power) after fitting the basic occupancy model compared to the MacKenzie–Bailey test (Figs. 1, 2 solid black line vs. dashed black line). The permutation join count test had higher power than either of the other two tests (Figs. 1, 2 dotted black lines), but again, this test only explores correlation in binary data prior to fitting a model and assumes no heterogeneity in the probability of detection. When correlation among detection probabilities was appropriately modeled with a Markov model, the MacKenzie–Bailey and join count chi‐square tests both displayed nominal test size as expected (red lines close to specified *α* = 0.05 for both tests; Figs. 1, 2). Overall, the join count chi‐square test was more sensitive (compared to the MacKenzie–Bailey test) when we defined neighbors as adjacent temporal replicates (Fig. 2), which is consistent with the Markov model used to simulate data (Fig. 1).

### Hoary bat and big brown bat acoustic data analyses

Using the Markov model, the estimated coefficient of the first‐order Markov structure covariate was 1.88 (SE = 0.24) for the hoary bat (LACI) and 1.88 (SE = 0.33) for the big brown bat (EPFU) datasets (Table 1), suggesting the presence of correlated detections for both species. For LACI, baseline occupancy (sample units without agriculture) was estimated to be 0.91 (95% CI from 0.75 to 0.97) using the basic model and 0.95 (95% CI from 0.72 to 0.99) using the Markov model. For EPFU, baseline occupancy was estimated to be 0.58 (95% CI from 0.42 to 0.73) using the basic model and 0.65 (95% CI from 0.43 to 0.82) using the Markov model. These were similar to our simulation findings with wider intervals using the Markov model for estimation in the presence of correlated detections. For both of these species, neither model provided evidence that the probability of occupancy differed for sample units with surrounding agriculture lands compared to those with no surrounding agriculture (all coefficients within 1 SE of zero, Table 1).

**Table 1:** Estimated coefficients (with standard errors) for basic and Markov occupancy models fit to hoary bat (LACI) and big brown bat (EPFU) datasets

	LACI		EPFU	
	Basic	Markov	Basic	Markov
Occupancy				
Intercept	2.27 (0.61)	3.01 (1.06)	0.34 (0.34)	0.61 (0.45)
Ag	0.23 (1.23)	0.53 (3.13)	−0.41 (0.60)	−0.59 (0.67)
Detection				
Intercept	−0.26 (0.13)	−1.13 (0.17)	−0.75 (0.20)	−1.54 (0.25)
Water2	0.54 (0.22)	0.32 (0.20)	0.44 (0.28)	0.32 (0.27)
Water3	0.96 (0.29)	0.71 (0.27)	0.38 (0.37)	0.25 (0.36)
Water4	0.98 (0.47)	0.35 (0.41)	1.82 (0.88)	0.79 (0.96)
Date	0.005 (0.003)	0.002 (0.003)	−0.004 (0.005)	−0.005 (0.005)
Mark	–	1.88 (0.24)	–	1.88 (0.33)

The first set of coefficients is for model terms associated with the occupancy (*ψ*) parameter (“Ag” indicator for sample unit surrounded by agricultural land). The second set of coefficients explained heterogeneity in detection probabilities (*p*) associated with different revisits. The water source factor used to model detection probabilities had four levels: creeks and streams (baseline), lakes and ponds (“Water2”), marshes and sloughs (“Water3”), and other (typically man‐made) water features (“Water4”). The “Date” detection coefficient was for Julian date (mean centered). The “Mark” detection coefficient was for the first‐order Markov‐structured covariate indicating whether the previous visit had a detection or not. For both of these species, the estimated coefficient for the Markov parameter was more than two standard errors greater than zero.

Using the basic model for LACI data, the detection coefficients for water source suggested that detection probability for all other water sources (lakes/ponds, marshes/sloughs, other/man‐made) was greater than that for creeks and streams (coefficient estimates more than two SEs larger than zero, Table 1). The magnitude of all of these coefficients was reduced after including the first‐order Markov‐structured covariate. For this model, the estimated coefficients suggested that only detection for marshes and sloughs was greater than that of creeks and streams (estimate = 0.71, SE = 0.27, Table 1). Comparatively, for the EPFU data, only the man‐made (and “other”) water sources differed in detection probabilities from the baseline level of creeks and streams (estimate = 1.82, SE = 0.88, Table 1). For the Markov model with the EPFU data, there was no evidence that any of the estimated water feature coefficients differed from zero. For both of these species, neither model indicated Julian date was associated with the probability of detection (all estimates within two SEs of zero, Table 1).

Specifying the basic model for LACI data, the MacKenzie–Bailey test indicated little evidence of lack of fit (*P*‐value = 0.138) while the join count chi‐square tests provided strong evidence of lack of fit using both neighborhoods (*P*‐values <0.002 when neighbors were defined as all nights from a detector (JC1) and when defined as only adjacent nights at a detector (JC2); Table 2). Interestingly, even after accounting for serial correlation within the LACI data, there was still evidence of lack of fit based on the join count chi‐square tests, but the MacKenzie–Bailey test provided no evidence of inadequate fit. The inadequacy of the Markov model for this species could be the result of the presence of a more complicated correlation structure that was not accounted for using the first‐order detection covariate or another form of detection probability heterogeneity which resulted in more observed joins than expected under the fitted model. A more comprehensive analysis for LACI was beyond the scope of this paper.

**Table 2:** *P*‐values from the MacKenzie–Bailey (MB) and join count chi‐square(JC1: neighbor definition 1, JC2: neighbor definition 2) tests from basic and Markov occupancy models fit to hoary bat (LACI) and big brown bat (EPFU) datasets. Available covariates for *p* and *ψ* were included in each model. These data were collected for acoustic surveys of wildlife refuges in the Pacific northwest

Species	Basic model			Markov model		
	MB	JC1	JC2	MB	JC1	JC2
LACI	0.138	<0.002	<0.002	0.774	0.012	0.026
EPFU	0.984	0.030	0.020	0.268	0.236	0.202

For the EPFU data, when using the basic model, the MacKenzie–Bailey test also provided no evidence of lack of fit (*P*‐value = 0.984, Table 2) while both join count chi‐square tests provided strong evidence for inadequate fit (JC1 *P*‐value = 0.02, JC2 *P*‐value = 0.03, Table 2). After serial correlation in EPFU data was accounted for using the Markov model, all tests provided no evidence of inadequate fit (*P*‐values > 0.2, Table 2).

## Discussion

As the number of occupancy modeling approaches and applications (e.g., Bailey et al. 2014; Kery and Royle 2016) grows, so does the need to provide accessible guidance and tools to practitioners for model assessment. Technological advances are enabling survey designs that use serial deployments of remote data loggers which accumulate large datasets of correlated observations. While this growing capacity allows researchers to encounter rare and cryptic organisms and to save money (e.g., reduced travel costs), these datasets potentially violate a central assumption of independent replicate observations from a sample unit. The use of model comparison metrics such as AIC is insufficient for detecting violations of such model assumptions. Our novel join count chi‐square test provides a targeted assessment of whether detections are correlated within a sample unit and strengthens goodness‐of‐fit procedures for an emerging and ever increasing type of occupancy survey design. Importantly, although we demonstrate our tool with two specific single‐season models, it is widely applicable to any occupancy model structure.

We found that our test had consistently higher power than the MacKenzie and Bailey (2004) goodness‐of‐fit test to detect lack of fit when basic occupancy models were inadequate because of correlated detections within a sample unit. When the neighborhood definition was aligned with the generating temporal process (first‐order Markov), the join count chi‐square test displayed higher power compared to a less restrictive temporal neighborhood definition. For both tests, power increased with increasing strength of correlation, but was less obviously responsive to the number of spatial and temporal replicates within a sample unit. These results emphasize that the MacKenzie–Bailey test may not always be optimal for identifying situations when data are inconsistent with the assumption of independent replicate surveys. This finding could be due to the fact that the MacKenzie–Bailey test is an omnibus assessment of model–data agreement. Our join count chi‐square test, on the other hand, focuses on whether the fitted model adequately captures correlation among detections within a sample unit. Furthermore, the flexible neighborhood definition in our join count chi‐square test allows for an investigation into multiscale temporal and spatial processes within an occupancy modeling framework. In this way, our tool can help guide practitioners by providing an additional, more specific test for assessing whether using observations gathered close together in time or space as independent revisits to a sample unit is inappropriate.

Identifying whether observations of detection/nondetection clustered in time or space are independent is important for ensuring estimates of occupancy are unbiased. As documented by others (Hines et al. 2010), our simulations showed that unmodeled correlation can lead to biased occupancy estimates. We found bias increased with increasing serial correlation and was greatest for within‐season revisit designs without an additional source of independent observations. For instance, we found that a basic occupancy model provided reliable inferences when correlation was low to moderate (*p*_1_ − *p*_0_ ≤ 0.6) under the proposed within‐season revisit design for the NABat (Loeb et al. 2015), four detectors with four night deployments within a sample unit. However, revisit designs with less spatial or temporal replicates produced biased estimates using the basic model. Importantly, the Markov model yielded unbiased estimates, except when correlation was very high (*p*_1_ − *p*_0_ = 0.8) and within‐season revisit designs consisted only of temporal replicates. While this work focused on bat surveys as a motivating example, many other practical survey designs used for occupancy estimation can lead to the potential for correlation among detections. For instance, Markov models were initially developed in order to analyze data collected along adjacent trail segments (Hines et al. 2010). Our simulations and assessments relied on the assumption of independent spatial replicates widely separated within an areal sample unit and Markov‐structured correlation among consecutive temporal replicates. This assumption may not be realistic for other survey designs (e.g., when detectors are placed along transects) and for other taxa. In these more complicated situations, the multiscale models of Nichols et al. (2008) might be more appropriate. Ultimately, any occupancy design which results in potentially correlated observations within a sample unit can benefit from an assessment of the independence assumption and our method provides such a diagnostic tool for use after fitting *any* occupancy model.

Over the last decade, formerly common species such as the little brown Myotis (*Myotis lucifugus*) and hoary bat have experienced unprecedented mortality rates and are now facing nontrivial extinction risks (Frick et al. 2010; Hayes 2013). One of the responses to these concerns has been the development of a plan for coordinated continental‐scale monitoring of bats, the NABat (Loeb et al. 2015). The scope and scale of this monitoring program and the elusive nature of bats (highly mobile and nocturnal) make accounting for imperfect detection while estimating occupancy, an important component of this program. Pilot testing of acoustic surveys conducted for four consecutive nights has begun in several jurisdictions, including the U.S. Fish and Wildlife Service (FWS). Using automated detection devices over consecutive nights raises the question of whether nightly surveys can still be considered independent revisits; our study suggests they may not be (e.g., hoary bat and big brown bat had “Mark” covariates >0 in Table 1). When temporal correlation is strong, a more efficient approach may be to utilize multiple spatial replicates dispersed widely within areal sample units to cover a range of available habitats with single‐night surveys. Replicating these single‐night surveys widely separated in time (e.g., monthly) within a defined season could avoid temporal correlation and provide more robust inferences.

Our analyses of hoary bat and big brown bat datasets provided strong evidence for correlated detections among consecutive nights. Similar to the results of our simulation study, the join count chi‐square test identified lack of fit of the basic model which was not found using the MacKenzie–Bailey test for both species. Accounting for correlation by way of the Markov model appeared effective for the big brown bat, but this approach will likely not be sufficient for all species (e.g., hoary bat). This comparison underscores the importance of assessing model fit. While the Markov model is expected to effectively account for correlation in most datasets, other models may be more appropriate for some bat species. This emphasizes that while the breadth of occupancy model extensions provides the potential to account for various survey designs and assumption violations, the use of a more sophisticated model does not guarantee adequate model fit to real data. Therefore, model assessment tools, such as our join count chi‐square test, need to be utilized in order to evaluate models and understand potential deficiencies.

## Conflict of Interest

None declared.

## Supporting information


**Data S1.** R code to perform the MacKenzie–Bailey and join count chi‐square tests using single‐season, single‐species occupancy models with covariates. Included is an example with simulated data.

**Appendix S1.** Empirical Assessment of Spatial and Temporal Correlation in Acoustic Bat Data.
**Appendix S2.** Neighborhood and join count calculation examples.
**Appendix S3.** Simulation results.
