# Supplementary material for: A goodness‐of‐fit test for occupancy models with correlated within‐season revisits
Source: Ecol Evol. 2016 Jul 5;6(15):5404–15. doi: 10.1002/ece3.2292 (PMC4984513; doi:10.1002/ece3.2292)
Supplement: Supplementary file 1 — Data S1. R code to perform the MacKenzie–Bailey and join count chi‐square tests using single‐season, single‐species occupancy models with covariates. Included is an example with simulated data. [file ECE3-6-5404-s001.zip › ece32292-sup-0004-DataS1/ece32292-sup-0004-DataS1.docx]

The R scripts in this folder provide an example of the methods presented in the paper. The following packages are required to run this code:

dplyr, tidyr, compiler, inline, Rcpp, RcppArmadillo, and metaSEM

Rtools also needs to be installed on the machine. Directions for how to do this can be found on the Rtools website.

To run the code, follow the instructions in the ‘DataS1A.R’ script. The ‘DataS1B.R’ script contains functions used to perform the tests, but is run in the ‘DataS1A.R’ script using the source() function.

The example utilizes a simulated dataset so that the estimates and results of the test can be compared to the ‘truth’.

R Package References:

Cheung, M.W.L. (2015) metaSEM: An R Package for Meta-Analysis using Structural Equation Modeling. *Frontiers in Psychology*, 5, 1521. URL <http://journal.frontiersin.org/Journal/10.3389/fpsyg.2014.01521/abstract> DOI 10.3389/fpsyg.2014.01521

Eddelbuettel, D. & Francois, R. (2001) Rcpp: Seamless R and C++ Integration. *Journal of Statistical Software*, 40, 1-18. URL <http://www.jstatsoft.org/v40/i08/>.

Eddelbuettel, D. & Sanderson, C. (2014) RcppArmadillo: Accelerating R with high-performance C++ linear algebra. *Computational Statistics and Data Analysis*, 71, 1054-1063. URL <http://dx.doi.org/10.1016/j.csda.2013.02.005>

Skylar, O., Murdoch, D., Smith, M., Eddelbuettel, D., Francois, R. & Soetaert, K. (2015). *inline: Functions to Inline C, C++, Fortran Function Calls from R*. R package version 0.3.14. URL <http://CRAN.R-project.org/package=inline>

Wickham, H. (2015) *tidyr: Easily Tidy Data with 'spread()' and 'gather()' Functions*. R package version 0.3.1. URL <http://CRAN.R-project.org/package=tidyr>

Wickham, H. & Francois, R. (201). *dplyr: A Grammar of Data Manipulation*. R package version 0.4.3. URL <http://CRAN.R-project.org/package=dplyr>
