# Supplementary material for: A goodness‐of‐fit test for occupancy models with correlated within‐season revisits
Source: Ecol Evol. 2016 Jul 5;6(15):5404–15. doi: 10.1002/ece3.2292 (PMC4984513; doi:10.1002/ece3.2292)
Supplement: Supplementary file 2 — Appendix S1. Empirical Assessment of Spatial and Temporal Correlation in Acoustic Bat Data. Appendix S2. Neighborhood and join count calculation examples. Appendix S3. Simulation results. [file ECE3-6-5404-s002.docx]

**Appendix**

**Appendix S1. Empirical Assessment of Spatial and Temporal Correlation in Acoustic Bat Data**

In this appendix, we empirically assess evidence for lack of independence among spatial and temporal replicate bat acoustic surveys prior to fitting an occupancy model (therefore, under the assumption that detection and occupancy probabilities are constant across sample units for a given species). This approach was used as a tool to explore potential types of correlation among revisits for bat acoustic data. We conducted this analysis as part of our goodness-of-fit test methodological development, but report these results separately as they are not central to the main results. We used the same call file data identified to bat species that were used for the empirical portion of the present study (Barnett 2014), but include here results for all species identified, rather than just the two species of focus used to motivate the goodness-of-fit methodology (hoary bat and big brown bat).

The species evaluated are as follows: pallid bat (ANPA), Townsend’s big-eared bat (COTO), big brown bat (EPFU), hoary bat (LACI), silver-haired bat (LANO), California Myotis (MYCA), western small-footed Myotis (MYCI), long-eared Myotis (MYEV), little brown bat (MYLU), fringed Myotis (MYTH), long-legged Myotis (MYVO), Yuma Myotis (MYYU), and canyon bat (PAHE).

For each species, only data from sample units which had detections (naively occupied) were used for calculating the permutation join count test (described in **Methods** section). The neighborhoods for each test were defined as: 1) (test 1) any pair of observations within the same sample unit and from the same year (pooled spatio-temporal correlation); 2) (test 2) any pair of observations from different stations within the same sample unit on the same night (i.e. spatial correlation among spatial replicates [stations]); 3) (test 3) observations from the same detector on consecutive nights during serial deployments (i.e. temporal correlation within a station). The p-values for all three tests are based on the BB (detection-detection) permutation tests (*joincount.mc* function) using the spdep package in R (Bivand and Piras 2015).

The permutation join count tests suggested that correlation among serial deployments was common (Table A1). For most species, there was evidence of correlation between pairs of observations within a sample unit (test 1) or when neighbors were defined as temporal replicates or nights surveyed at the same spatial location (station) within a sample unit (test 3). There was almost no evidence for correlation among detector stations (spatial replicates; test 2) except for the sparsely recorded Townsend’s big-eared bat (COTO). These results support our assumption, relied upon for methodological development purposes (but not ruled out as a potential concern, in general), that spatial replicates within sample units could plausibly be considered independent, but that consecutive nightly replicates from the same station (detector location) could not be.

**Table A1**. Permutation join count p-values for each species from the three tests which each used different neighbor definitions. Test 1 treated all observations within the same sample unit as neighbors, test 2 defined spatial replicates as neighbors within the same night, test 3 treated nights at the same detector as neighbors. Light gray shading highlights p-values < 0.05.

| Species | Test 1 | Test 2 | Test 3 |
| --- | --- | --- | --- |
| ANPA | 0.360 | 0.402 | 0.364 |
| COTO | 0.001 | 0.001 | 0.006 |
| EPFU | 0.593 | 0.774 | 0.001 |
| LACI | 0.001 | 0.114 | 0.001 |
| LANO | 0.001 | 0.476 | 0.001 |
| MYCA | 0.471 | 0.758 | 0.001 |
| MYCI | 0.001 | 0.127 | 0.001 |
| MYEV | 0.008 | 0.094 | 0.001 |
| MYLU | 0.001 | 0.234 | 0.001 |
| MYTH | 0.780 | 0.806 | 0.046 |
| MYVO | 0.627 | 0.750 | 0.001 |
| MYYU | 0.156 | 0.703 | 0.001 |
| PAHE | 0.656 | 0.862 | 0.001 |

**Appendix S2. NEIGHBORHOOD AND JOIN COUNT CALCULATION EXAMPLES**

Example 1:

First we consider a within-season revisit design with two spatial replicates (e.g. in our motivating example two bat detectors located at different stations within an areal sample unit with J=2) and four temporal replicates (in our case, four consecutive night surveys with K=4) for a single sample unit (Table B1). For this within-season revisit design and using neighborhood definition 1 (all nights at the same detector are considered neighbors), there are 12 unique neighbor pairs since h_11_/h_12_ is not a different pair from h_12_/h_11_, for instance (Figure B1). Note that all of the diagonals are 0’s because an observation cannot be a neighbor with itself and for simplicity, only the upper triangle of the matrix is shown because it is symmetric. Observations from different detectors (shaded) cannot be neighbors based on the definitions we used. Using our second neighbor definition (adjacent nights at the same detector are considered neighbors) we have only six unique neighbor pairs in total (Figure B2). The neighbor definition and resulting number of pairs will determine the possible BB values used in the test statistic. For either definition, the number of joins for a sample unit is determined by the number of unique neighbor pairs (i.e. observations that have a 1 in the upper triangle of the neighbor matrix Figure B1 and B2) where both have a detection (1) recorded.

To illustrate the number of joins for a particular detection history, consider observing the history h=0011 1010 with two spatial replicates and four temporal replicates. Based on neighborhood definition 2 (adjacent nights at the same detector are considered neighbors), this history has 1 join because h_13_ and h_14_ are the only neighbors that both had an observed detection of the species. Since h_14_ and h_21_ are at different detectors and therefore not neighbors, this pair does not contribute a join to this summary measure even though these detections appear to be consecutive in the history above. This same approach would be used to calculate the number of joins for other histories and/or other neighbor definitions. The number of joins can be calculated with the matrix multiplication 0.5*(**h N h**^T^), where **h** is a row vector for the detection history and **N** is the entire neighbor matrix (including the lower triangle).

Example 2:

For another example, consider a within-season revisit design that is composed of only one spatial replicate (detector) with three temporal replicates (consecutive nights, J=1 and K=3), there are three total revisits (Table B2). Using neighbor definition 2 (adjacent nights at the same detector are considered neighbors); there are 2 unique neighbor pairs (Figure B3). In the case of neighbor definition 1 (all nights at the same detector are considered neighbors) there would be 3 unique neighbor pairs.

Assuming this simple revisit design structure (Table B2), we demonstrate some of the basic calculations for the join count (JC) χ^2^ test for a small hypothetical dataset with 10 total sample units. There are eight possible detection histories (column 1, Table B3) under this revisit design and for illustration we assume five of the possible outcomes were observed (column 2, Table B3). For a *single* sample unit, the calculation for the probability of a given detection history under our basic (single-species, single-season) model follows from MacKenzie *et al.* (2006; column 3, Table B3) and a similar calculation can be done for the Markov model (column 4, Table B3). In each of these models, the probabilities associated with occupancy and detection are assumed to be constant across the different sample units. However, this is just an example and our method is applicable to models with variable occupancy and detection probabilities.

The information in Table B3 can be used to calculate the needed quantities for the JC χ^2^ test. First, note we are using definition 2 so there are two unique neighbor pairs at each sample unit under this design – h_11_/h_12_ and h_12_/h_13_ (Figure B3). Therefore, there is a maximum of two joins (both observations in both neighbor pairs are detections) possible. From Table B3, detection histories 1, 2, 3, 4, and 6 all correspond to 0 joins. Therefore, the observed number of sample units with 0 joins would be 4 + 1 + 0 + 0 + 0 = 5. Similarly, histories 5 and 7 correspond to 1 join so there were 3 sample units observed with a BB value of 1; history 8 corresponds to 2 joins and there were 2 observed sample units with BB=2.

The expected number of sample units with a BB value can be found by adding together the probabilities of the detection histories associated with that number of joins and, with no covariates, multiplying by the total number of sample units. With occupancy or detection probabilities varying by sample unit, the corresponding probabilities are summed across all sample units as described by MacKenzie & Bailey (2004). For the example here, the expected number of sites with 1 join under the basic model is then found with

$$10*\sum_{h=1}^{8} I\left( \mathrm{joins}\left( h \right)=1 \right)*\hat{P}r \left( h \right)=10*\left\{ \hat{\psi}\left( 1-\hat{p} \right)\hat{p}^{2}+\hat{\psi}\left( 1-\hat{p} \right)\hat{p}^{2} \right\},$$

where the detection history probabilities added here correspond to 5 and 7 from Table B3 which both have 1 join. Similarly, for the Markov model, the same idea can be used to calculate the expected number of sites with a single join:

$$10*\sum_{h=1}^{8} I\left( \mathrm{joins}\left( h \right)=1 \right)*\hat{P}r \left( h \right)=10*\left\{ \hat{\psi}\left( 1-\hat{p}_{m} \right)\hat{p}_{0}\hat{p}_{1}+\hat{\psi}\hat{p}_{m}\hat{p}_{1}\left( 1-\hat{p}_{1} \right) \right\}.$$

This process would be repeated for the other values (0 and 2) that the number of joins could take on in this example to calculate all the E_BB_ values for each model. Using example 2 and neighbor definition 2, example calculations for the basic model (Tables B4 and B5) and the Markov model (Tables B6 and B7) are shown using some possible (made up) parameter estimates.

Under either model, the JC χ^2^ test statistic can be calculated using the observed and expected join counts by

$$\sum_{\mathrm{BB}} \frac{(O_{\mathrm{BB}}-E_{\mathrm{BB}})^{2}}{E_{\mathrm{BB}}}.$$

The same general process would be used to perform the test using the different neighbor definition and (or) for the within-season revisit design from example 1.

The previous example calculations assume that there is no heterogeneity in the probabilities of detection or occupancy among sites. This assumption allowed the expected counts under the model to be found by multiplying the number of sample units (10) by the probability of a particular number of joins. Therefore, to adjust this calculation for models that allow *ψ* and *p* to vary we add the estimated probabilities across all sample units. For instance, for a basic model the calculation of the expected number of sites with 1 join is now

$$\sum_{i=1}^{10} \sum_{h=1}^{8} I\left( \mathrm{joins}\left( h_{i} \right)=1 \right)*\hat{P}r \left( h_{i} \right)=\sum_{i=1}^{10} \left\{ \hat{\psi}_{i}\left( 1-\hat{p}_{i1} \right)\hat{p}_{i2}\hat{p}_{i3}+\hat{\psi}_{i}\hat{p}_{i1}\hat{p}_{i2}\left( 1-\hat{p}_{i3} \right) \right\},$$

since, again, histories 5 (011) and 7(110) both have a single join. Here, $\hat{p}_{ik}$ is the estimated probability of detection for site *i* and revisit *k* (the subscript for spatial units was dropped because there is only 1 in this example). Now the probabilities of occupancy and detection are estimated uniquely for each sample unit and the overall expected number of sample units with 1 join is found by summing these probabilities across all of the sample units. A similar calculation would be done for the Markov model, now with the parameters estimated under that model. Again, for either model the approach is repeated for all the other possible join (BB) values under the particular neighbor definition and sampling scheme.

If the design is unbalanced (i.e. J and/or K not all equal for every sample unit) the unique patterns of J and K define different cohorts. For each cohort, the observed and expected joins (BB) would be calculated separately and then combined in the overall test statistic by summing over all cohorts. These general steps are outlined by MacKenzie & Bailey (2004) using unique detection histories and here we apply the approach to the number of joins at a sample unit. The same approach could be generalized for any summary measure of interest.

Table B1: Within-season revisit design for a single sample unit assuming two spatial replicates (detectors) and four temporal replicates (nights) with h_jk_ representing elements within a detection history matrix with potential values of 0=no detection or 1=detection.

|  | **Night 1** | **Night 2** | **Night 3** | **Night 4** |
| --- | --- | --- | --- | --- |
| **Detector 1** | h_11_ | h_12_ | h_13_ | h_14_ |
| **Detector 2** | h_21_ | h_22_ | h_23_ | h_24_ |

Table B2: Within-season revisit design for a single sample unit with only one spatial replicate (detector) and three temporal replicates (nights) with h_jk_ representing elements within a detection history matrix with potential values of 0=no detection or 1=detection.

|  | **Night 1** | **Night 2** | **Night 3** |
| --- | --- | --- | --- |
| **Detector 1** | h_11_ | h_12_ | h_13_ |

Table B3: Possible detection histories for a hypothetical dataset with three temporal replicates (as in Example 2 Table B2), associated probabilities under the basic and Markov models, and number observed in a hypothetical dataset.

|  | History(h) | # Observed | Pr(h)-Basic | Pr(h)-Markov |
| --- | --- | --- | --- | --- |
| 1 | 0 0 0 | 4 | *Ψ*(1-*p*)^3^ + (1- *Ψ*) | *Ψ*(1-*p*_m_)(1-*p*_0_)^2^ + (1- *Ψ*) |
| 2 | 0 0 1 | 1 | *Ψ*(1-*p*)^2^*p* | *Ψ*(1-*p*_m_)(1-*p*_0_)*p*_0_ |
| 3 | 0 1 0 | 0 | *Ψ*(1-*p*)^2^*p* | *Ψ*(1-*p*_m_)*p*_0_(1-*p*_1_) |
| 4 | 1 0 0 | 0 | *Ψ*(1-*p*)^2^*p* | *Ψp*_m_(1-*p*_1_)(1-*p*_0_) |
| 5 | 0 1 1 | 2 | *Ψ*(1-*p*)*p^2^* | *Ψ*(1-*p*_m_)*p*_0_*p*_1_ |
| 6 | 1 0 1 | 0 | *Ψ*(1-*p*)*p^2^* | *Ψp*_m_(1-*p*_1_)*p*_0_ |
| 7 | 1 1 0 | 1 | *Ψ*(1-*p*)*p^2^* | *Ψp*_m_*p*_1_(1-*p*_1_) |
| 8 | 1 1 1 | 2 | *Ψp^3^* | *Ψp*_m_(*p*_1_)^2^ |

Table B4: Possible detection histories for a revisit design with only three temporal replicates within a sample unit (as in Example 2 Table B2), associated model probabilities, and possible associated estimated probabilities under the basic model.

|  | History(h) | Pr(h)-Basic | $\hat{\psi}$=0.65, $\hat{p}$=0.4 |
| --- | --- | --- | --- |
| 1 | 0 0 0 | *Ψ*(1-*p*)^3^ + (1- *Ψ*) | 0.65(0.6)^3^ + (0.35)= 0.4904 |
| 2 | 0 0 1 | *Ψ*(1-*p*)^2^*p* | 0.65(0.6)^2^0.4 = 0.0936 |
| 3 | 0 1 0 | *Ψ*(1-*p*)^2^*p* | 0.65(0.6)^2^0.4 = 0.0936 |
| 4 | 1 0 0 | *Ψ*(1-*p*)^2^*p* | 0.65(0.6)^2^0.4 = 0.0936 |
| 5 | 0 1 1 | *Ψ*(1-*p*)*p^2^* | 0.65(0.6)(0.4)^2^ = 0.0624 |
| 6 | 1 0 1 | *Ψ*(1-*p*)*p^2^* | 0.65(0.6)(0.4)^2^ = 0.0624 |
| 7 | 1 1 0 | *Ψ*(1-*p*)*p^2^* | 0.65(0.6)(0.4)^2^ = 0.0624 |
| 8 | 1 1 1 | *Ψp^3^* | 0.65(0.4)^3^ = 0.0416 |

Table B5: Calculations of the observed and expected join counts using the basic model and possible parameter estimates for example 2 and neighbors defined as adjacent nights.

| BB | O_BB_ | E_BB_ |
| --- | --- | --- |
| 0 | 5 | 10*(0.4904 + 3*0.0936 + 0.0624) = 8.336 |
| 1 | 3 | 10*(2*0.0624)=1.248 |
| 2 | 2 | 10*(0.0416) = 0.416 |

Table B6: Possible detection histories, associated model probabilities, and possible associated estimated probabilities under the Markov model for example 2.

|  | History(h) | # Observed | Pr(h)-Markov | $\hat{\psi}$=0.75, $\hat{p}_{0}$=0.3, $\hat{p}_{1}$=0.6 |
| --- | --- | --- | --- | --- |
| 1 | 0 0 0 | 4 | *Ψ*(1-*p*_m_)(1-*p*_0_)^2^ + (1- *Ψ*) | 0.46 |
| 2 | 0 0 1 | 1 | *Ψ*(1-*p*_m_)(1-*p*_0_)*p*_0_ | 0.09 |
| 3 | 0 1 0 | 0 | *Ψ*(1-*p*_m_)*p*_0_(1-*p*_1_) | 0.05142857 |
| 4 | 1 0 0 | 0 | *Ψp*_m_(1-*p*_1_)(1-*p*_0_) | 0.09 |
| 5 | 0 1 1 | 2 | *Ψ*(1-*p*_m_)*p*_0_*p*_1_ | 0.07714286 |
| 6 | 1 0 1 | 0 | *Ψp*_m_(1-*p*_1_)*p*_0_ | 0.03857143 |
| 7 | 1 1 0 | 1 | *Ψp*_m_*p*_1_(1-*p*_1_) | 0.07714286 |
| 8 | 1 1 1 | 2 | *Ψp*_m_(*p*_1_)^2^ | 0.1157143 |

Table B7: Calculations of the observed and expected join counts using the Markov model and possible parameter estimates for example 2 with neighbors defined as adjacent nights.

| BB | O_BB_ | E_BB_ |
| --- | --- | --- |
| 0 | 5 | 10*(0.46 + 2*0.09 + 0.05142857+0.03857143) = 7.3 |
| 1 | 3 | 10*(2*0.07714286)=1.542857 |
| 2 | 2 | 10*(0.1157143) = 1.157143 |

|  | h_11_ | h_12_ | h_13_ | h_14_ | h_21_ | h_22_ | h_23_ | h_24_ |
| --- | --- | --- | --- | --- | --- | --- | --- | --- |
| h_11_ | 0 | 1 | 1 | 1 | 0 | 0 | 0 | 0 |
| h_12_ | - | 0 | 1 | 1 | 0 | 0 | 0 | 0 |
| h_13_ | - | - | 0 | 1 | 0 | 0 | 0 | 0 |
| h_14_ | - | - | - | 0 | 0 | 0 | 0 | 0 |
| h_21_ | - | - | - | - | 0 | 1 | 1 | 1 |
| h_22_ | - | - | - | - | - | 0 | 1 | 1 |
| h_23_ | - | - | - | - | - | - | 0 | 1 |
| h_24_ | - | - | - | - | - | - | - | 0 |

Figure B1. Visualization showing which pairs of revisits within a detection history matrix composed of four nightly surveys at two detectors (Table B1) are considered neighbors under definition 1 of all nights at the same detector are neighbors. Only the upper triangle is displayed since the matrix is symmetric. Observations considered neighbors are denoted by 1 and those which are not neighbors are denoted by 0.

|  | h_11_ | h_12_ | h_13_ | h_14_ | h_21_ | h_22_ | h_23_ | h_24_ |
| --- | --- | --- | --- | --- | --- | --- | --- | --- |
| h_11_ | 0 | 1 | 0 | 0 | 0 | 0 | 0 | 0 |
| h_12_ | - | 0 | 1 | 0 | 0 | 0 | 0 | 0 |
| h_13_ | - | - | 0 | 1 | 0 | 0 | 0 | 0 |
| h_14_ | - | - | - | 0 | 0 | 0 | 0 | 0 |
| h_21_ | - | - | - | - | 0 | 1 | 0 | 0 |
| h_22_ | - | - | - | - | - | 0 | 1 | 0 |
| h_23_ | - | - | - | - | - | - | 0 | 1 |
| h_24_ | - | - | - | - | - | - | - | 0 |

Figure B2: Visualization of which revisits within a detection history matrix composed of four nightly surveys at two detectors (Table B1) are considered neighbors under definition 2 (adjacent nights at the same detector are considered neighbors). Only the upper triangle is displayed since the matrix is symmetric. Observations considered neighbors are denoted by 1 and those which are not neighbors are denoted by 0.

|  | h_11_ | h_12_ | h_13_ |
| --- | --- | --- | --- |
| h_11_ | 0 | 1 | 0 |
| h_12_ | - | 0 | 1 |
| h_13_ | - | - | 0 |

Figure B3: Visualization of which revisits within a detection history matrix composed of three nightly surveys and one detector (Table B2) are considered neighbors under definition 2 (adjacent nights at the same detector are considered neighbors). Only the upper triangle is displayed since the matrix is symmetric. Observations considered neighbors are denoted by 1 and those which are not neighbors are denoted by 0. Notice using a neighbor definition of all nights from the same detector would switch the pair (h_11_ h_13_) from 0 (not neighbors) to a 1.

**Appendix S3. SIMULATION RESULTS**

Table C1: Means of ψ estimates and the coverage (cov.) of 95% confidence intervals from basic and Markov occupancy models (which converged) fit to 500 simulated datasets for four spatial, four temporal; one spatial, 16 temporal; four spatial, two temporal; and one spatial, eight temporal revisit scenarios with different levels of correlation (p_1_-p_0_) among detections. The simulated probability of occupancy (ψ) in these scenarios was 0.75.

|  | Four spatial, four temporal | | | | One spatial, 16 temporal | | | |
| --- | --- | --- | --- | --- | --- | --- | --- | --- |
|  | Basic | | Markov | | Basic | | Markov | |
| Correlation(p_1_-p_0_) | Mean | Cov. | Mean | Cov. | Mean | Cov. | Mean | Cov. |
| 0.0 | 0.754 | 0.958 | 0.754 | 0.958 | 0.748 | 0.938 | 0.749 | 0.936 |
| 0.2 | 0.748 | 0.940 | 0.748 | 0.940 | 0.751 | 0.950 | 0.751 | 0.950 |
| 0.4 | 0.750 | 0.954 | 0.751 | 0.954 | 0.746 | 0.944 | 0.748 | 0.944 |
| 0.6 | 0.750 | 0.954 | 0.753 | 0.953 | 0.736 | 0.950 | 0.754 | 0.959 |
| 0.8 | 0.737 | 0.944 | 0.743 | 0.967 | 0.672 | 0.758 | 0.755 | 0.936 |
|  |  |  |  |  |  |  |  |  |
|  | Four spatial, two temporal | | | | One spatial, eight temporal | | | |
|  | Basic | | Markov | | Basic | | Markov | |
| Correlation(p_1_-p_0_) | Mean | Cov. | Mean | Cov. | Mean | Cov. | Mean | Cov. |
| 0.0 | 0.753 | 0.974 | 0.753 | 0.974 | 0.753 | 0.962 | 0.753 | 0.958 |
| 0.2 | 0.748 | 0.948 | 0.752 | 0.958 | 0.744 | 0.952 | 0.753 | 0.960 |
| 0.4 | 0.738 | 0.940 | 0.747 | 0.958 | 0.716 | 0.878 | 0.749 | 0.962 |
| 0.6 | 0.733 | 0.938 | 0.752 | 0.968 | 0.676 | 0.734 | 0.768 | 0.944 |
| 0.8 | 0.719 | 0.908 | 0.750 | 0.970 | 0.575 | 0.226 | 0.779 | 0.912 |

Table C2: Means of estimates, coverage rates of 95% confidence intervals, and 95% CI lengths for detection parameters using basic and Markov models on simulated datasets with different levels of correlation (p_1_-p_0_) among detections under scenarios with four spatial, four temporal and one spatial, 16 temporal replicates. For the basic models, coverage was the proportion of CIs that captured p_mean_=0.5. Results are shown only for converged models.

|  | | Four spatial, four temporal | | | One spatial, 16 temporal | | |
| --- | --- | --- | --- | --- | --- | --- | --- |
| MARKOV MODELS | Correlation(p_1_-p_0_) | Mean p_0_ | Coverage | CI Length | Mean p_0_ | Coverage | CI Length |
|  | 0.0 | 0.503 | 0.964 | 0.122 | 0.500 | 0.948 | 0.115 |
|  | 0.2 | 0.400 | 0.952 | 0.117 | 0.403 | 0.954 | 0.112 |
|  | 0.4 | 0.300 | 0.952 | 0.106 | 0.300 | 0.950 | 0.105 |
|  | 0.6 | 0.200 | 0.949 | 0.090 | 0.203 | 0.947 | 0.097 |
|  | 0.8 | 0.100 | 0.951 | 0.066 | 0.100 | 0.939 | 0.089 |
|  | Correlation(p_1_-p_0_) | Mean p_1_ | Coverage | CI Length | Mean p_1_ | Coverage | CI Length |
|  | 0.0 | 0.497 | 0.940 | 0.121 | 0.499 | 0.948 | 0.115 |
|  | 0.2 | 0.601 | 0.950 | 0.117 | 0.600 | 0.952 | 0.111 |
|  | 0.4 | 0.700 | 0.944 | 0.106 | 0.698 | 0.952 | 0.104 |
|  | 0.6 | 0.801 | 0.969 | 0.088 | 0.799 | 0.952 | 0.088 |
|  | 0.8 | 0.899 | 0.934 | 0.064 | 0.897 | 0.939 | 0.066 |
| BASIC MODELS | Correlation(p_1_-p_0_) | Mean p | Coverage | CI Length | Mean p | Coverage | CI Length |
|  | 0.0 | 0.500 | 0.942 | 0.080 | 0.500 | 0.964 | 0.080 |
|  | 0.2 | 0.501 | 0.910 | 0.080 | 0.502 | 0.890 | 0.080 |
|  | 0.4 | 0.500 | 0.866 | 0.080 | 0.500 | 0.828 | 0.080 |
|  | 0.6 | 0.505 | 0.794 | 0.080 | 0.509 | 0.700 | 0.081 |
|  | 0.8 | 0.508 | 0.732 | 0.081 | 0.556 | 0.366 | 0.084 |

Table C3: Means of estimates, coverage rates of 95% confidence intervals, and 95% CI lengths for detection parameters using basic and Markov models on simulated datasets with different levels of correlation (p_1_-p_0_) among detections under scenarios with four spatial, two temporal and one spatial, eight temporal replicates. For the basic models, coverage was the proportion of CIs that captured p_mean_=0.5. Results are shown only for converged models.

|  |  | Four spatial, two temporal | | | One spatial, eight temporal | | |
| --- | --- | --- | --- | --- | --- | --- | --- |
| MARKOV MODELS | Correlation(p_1_-p_0_) | Mean p_0_ | Coverage | CI Length | Mean p_0_ | Coverage | CI Length |
|  | 0.0 | 0.498 | 0.956 | 0.197 | 0.500 | 0.960 | 0.169 |
|  | 0.2 | 0.399 | 0.954 | 0.189 | 0.403 | 0.932 | 0.172 |
|  | 0.4 | 0.300 | 0.948 | 0.173 | 0.299 | 0.960 | 0.175 |
|  | 0.6 | 0.198 | 0.962 | 0.147 | 0.201 | 0.948 | 0.177 |
|  | 0.8 | 0.100 | 0.960 | 0.108 | 0.109 | 0.845 | 0.181 |
|  | Correlation(p_1_-p_0_) | Mean p_1_ | Coverage | CI Length | Mean p_1_ | Coverage | CI Length |
|  | 0.0 | 0.497 | 0.954 | 0.193 | 0.499 | 0.940 | 0.164 |
|  | 0.2 | 0.602 | 0.962 | 0.183 | 0.599 | 0.954 | 0.159 |
|  | 0.4 | 0.697 | 0.950 | 0.168 | 0.699 | 0.946 | 0.147 |
|  | 0.6 | 0.802 | 0.946 | 0.140 | 0.796 | 0.934 | 0.125 |
|  | 0.8 | 0.900 | 0.966 | 0.103 | 0.898 | 0.949 | 0.090 |
| BASIC MODELS | Correlation(p_1_-p_0_) | Mean p | Coverage | CI Length | Mean p | Coverage | CI Length |
|  | 0.0 | 0.498 | 0.948 | 0.114 | 0.500 | 0.928 | 0.114 |
|  | 0.2 | 0.503 | 0.926 | 0.115 | 0.506 | 0.916 | 0.115 |
|  | 0.4 | 0.503 | 0.912 | 0.116 | 0.520 | 0.772 | 0.117 |
|  | 0.6 | 0.512 | 0.880 | 0.116 | 0.555 | 0.534 | 0.119 |
|  | 0.8 | 0.520 | 0.806 | 0.117 | 0.660 | 0.048 | 0.122 |

Table C4: Average lengths of 95% confidence intervals for ψ after fitting basic and Markov models to simulated datasets under different revisit designs. Only results from converged models are shown.

|  | Four spatial, four temporal | | One spatial, 16 temporal | |
| --- | --- | --- | --- | --- |
| Correlation(p_1_-p_0_) | Basic | Markov | Basic | Markov |
| 0.0 | 0.234 | 0.234 | 0.236 | 0.235 |
| 0.2 | 0.236 | 0.236 | 0.235 | 0.235 |
| 0.4 | 0.235 | 0.235 | 0.236 | 0.237 |
| 0.6 | 0.235 | 0.237 | 0.239 | 0.245 |
| 0.8 | 0.239 | 0.248 | 0.253 | 0.372 |
|  |  |  |  |  |
|  | Four spatial, two temporal | | One spatial, eight temporal | |
| Correlation(p_1_-p_0_) | Basic | Markov | Basic | Markov |
| 0.0 | 0.237 | 0.237 | 0.237 | 0.237 |
| 0.2 | 0.238 | 0.239 | 0.239 | 0.244 |
| 0.4 | 0.241 | 0.245 | 0.245 | 0.271 |
| 0.6 | 0.242 | 0.251 | 0.253 | 0.394 |
| 0.8 | 0.245 | 0.264 | 0.265 | 0.659 |
