# Supplementary figures and images for: A goodness‐of‐fit test for occupancy models with correlated within‐season revisits
Source: Ecol Evol. 2016 Jul 5;6(15):5404–15. doi: 10.1002/ece3.2292 (PMC4984513; doi:10.1002/ece3.2292)

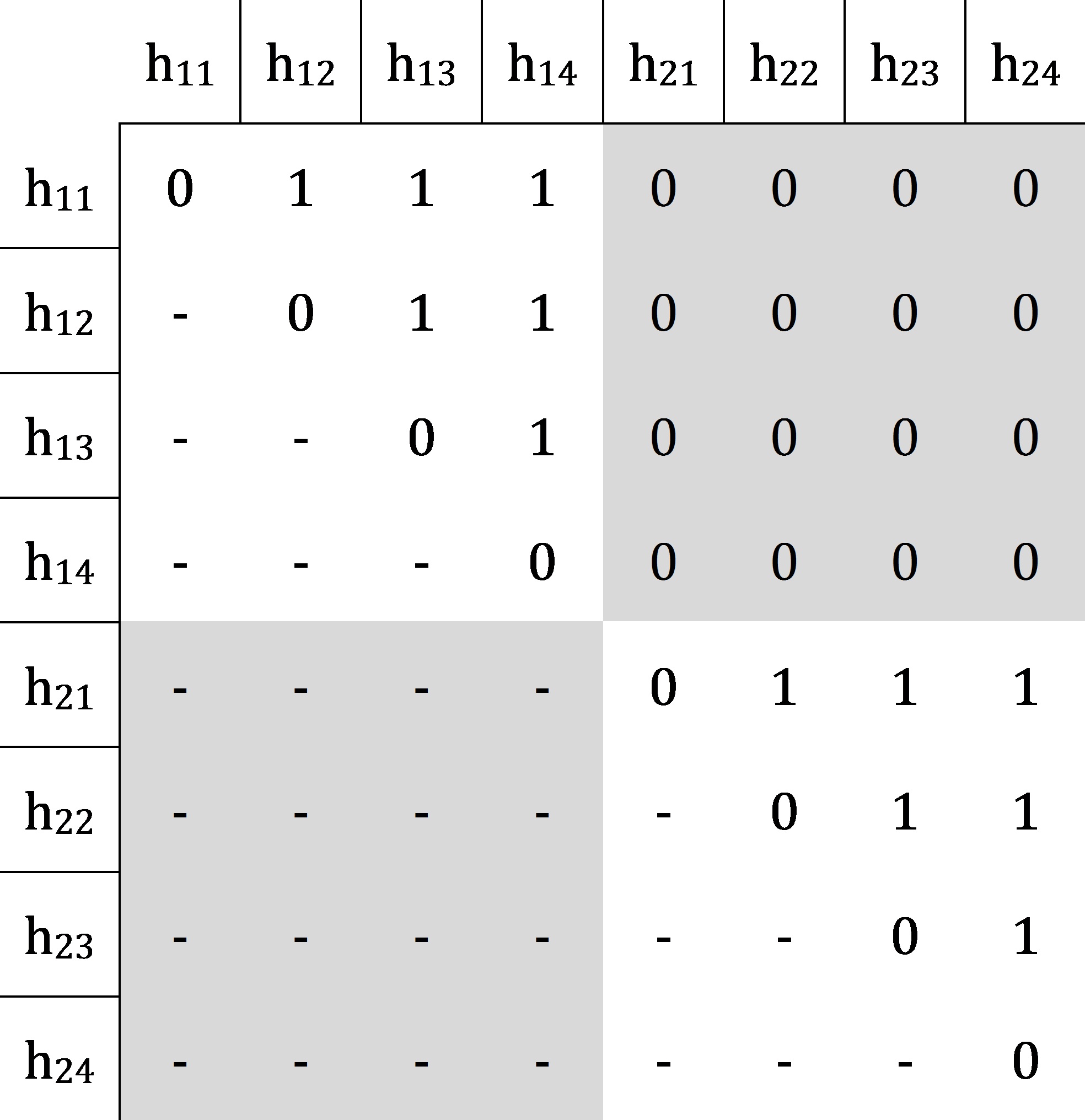

Supplement: Supplementary file 3 [file ECE3-6-5404-s003.png]

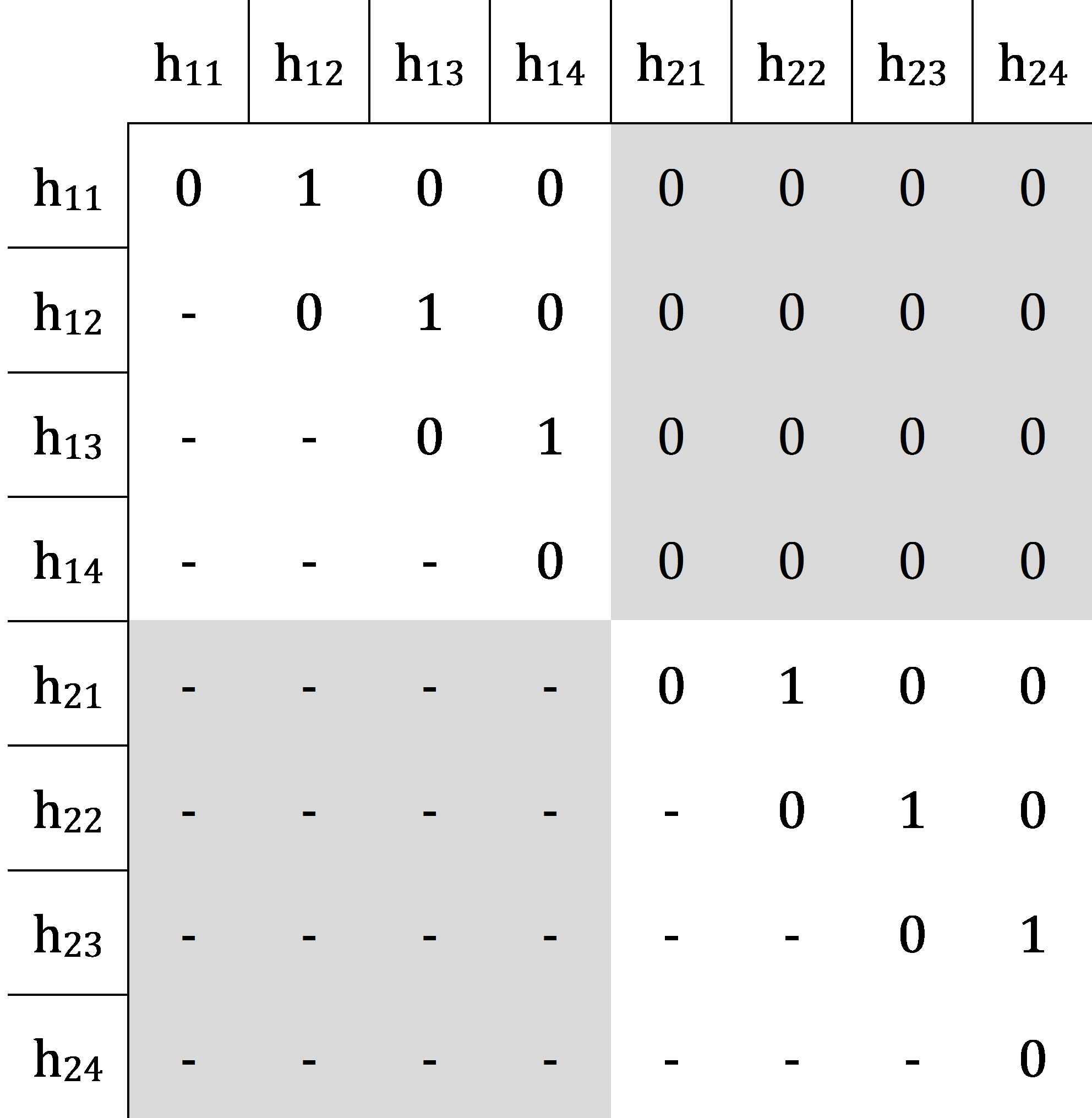

Supplement: Supplementary file 4 [file ECE3-6-5404-s004.png]

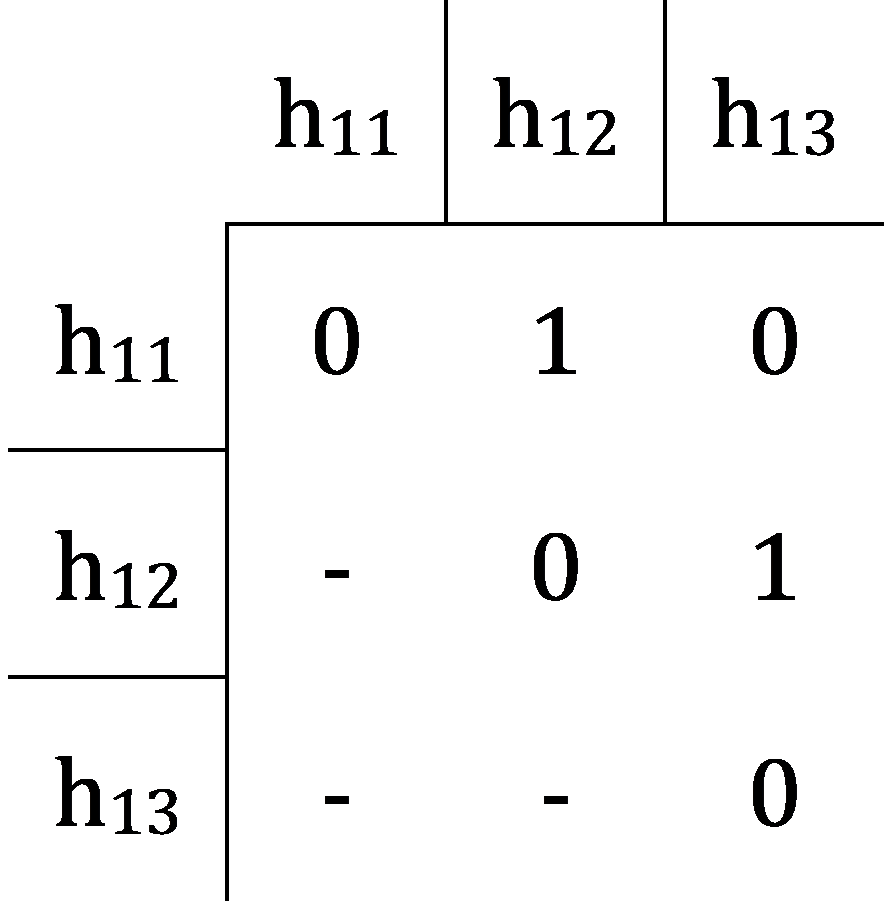

Supplement: Supplementary file 5 [file ECE3-6-5404-s005.png]
